# Supplementary material for: Alpha-Synuclein mRNA Level Found Dependent on L444P Variant in Carriers and Gaucher Disease Patients on Enzyme Replacement Therapy
Source: Biomolecules. 2023 Apr 3;13(4):644. doi: 10.3390/biom13040644 (PMC10135719; doi:10.3390/biom13040644)

**Supplementary Fifure S1.** Scatter plot of age (x-axis) and biomarkers (Lyso-Gb1, α-SNCA mRNA level, α-SNCA total protein concentration and oligomer concentration). Different point types indicate different subgroups.

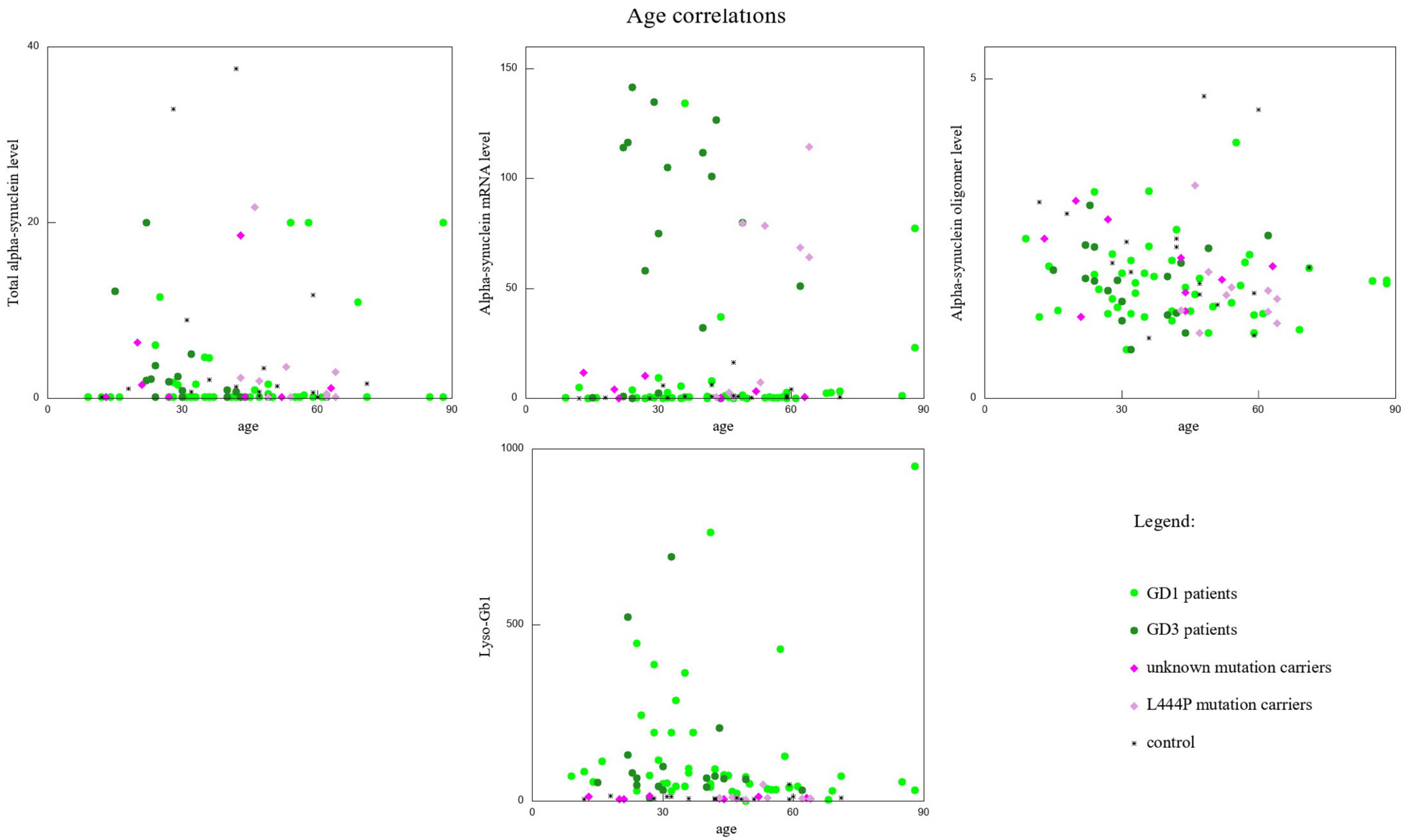

Supplement: Supplementary file 1 [file biomolecules-13-00644-s001.zip › Supplementary Fifure S1.pdf]
