# Supplementary material for: Alpha-Synuclein mRNA Level Found Dependent on L444P Variant in Carriers and Gaucher Disease Patients on Enzyme Replacement Therapy
Source: Biomolecules. 2023 Apr 3;13(4):644. doi: 10.3390/biom13040644 (PMC10135719; doi:10.3390/biom13040644)
Supplement: Supplementary file 1 [file biomolecules-13-00644-s001.zip › Supplementary Table S1.pdf]

Supplementary Table S1. Pearson linear correlation coefficient between the given marker (Lyso- Gb1, *SNCA* mRNA level, α-SNCA total protein concentration and oligomer concentration) and age in the following groups: GD1, GD3, unknown mutation carriers, L444P carriers, and control.

	**Lyso-Gb1**	**α-SNCA total protein concentration**	***SNCA* mRNA level**	**Oligomer concentration**
**GD1**	0,098275	0,289212	0,227364	-0,03859
**GD3**	-0,15565	-0,51458	0,068943	-0,05628
**Unknown mutation carriers**	-0,05349	0,034903	-0,60128	-0,38611
**L444P carriers**	-0,14434	-0,4052	0,741275	-0,33516
**Control**	0,223636	-0,1354	0,102784	-0,09648

α-SNCA – alpha-synuclein
GD – Gaucher disease
Lyso-Gb1 - Glucosylsphingosine
***SNCA* -** gene coding alpha-synuclein
